# Integrin β1, myosin light chain kinase and myosin IIA are required for activation of PI3K-AKT signaling following MEK inhibition in metastatic triple negative breast cancer

**DOI:** 10.18632/oncotarget.11525

**Published:** 2016-08-23

**Authors:** Cheolwon Choi, Junyeob Kwon, Sunyoung Lim, David M. Helfman

**Affiliations:** ^1^ Department of Biological Sciences, Korean Advanced Institute of Science and Technology, Daejeon, Korea

**Keywords:** myosin IIA, MLCK, integrin β1, MEK resistance, FAK

## Abstract

The effectiveness of targeted therapies against the Ras-ERK signaling pathway are limited due to adaptive resistance of tumor cells. Inhibition of the Ras-ERK pathway can result in activation of the PI3K-AKT pathway, thereby diminishing the therapeutic effects of targeting ERK signaling. Here we investigated the crosstalk between the Ras-ERK and PI3K-AKT pathways in MDA-MB-231 breast cancer cell lines that have a preference to metastasize to lung (LM2), brain (BrM2) or bone (BoM2). Inhibition of the Ras-ERK pathway reduced motility in both parental and BoM2 cells. In contrast, inhibition of the Ras-ERK pathway in BrM2 and LM2 cells resulted in activation of PI3K-AKT signaling that was responsible for continued cell motility. Analysis of the cross talk between Ras-ERK and PI3K-AKT signaling pathways revealed integrin β1, myosin light chain kinase (MLCK) and myosin IIA are required for the activation of PI3K-AKT following inhibition of the Ras-ERK pathway. Furthermore, feedback activation of the PI3K-AKT pathway following MEK suppression was independent of the epidermal growth factor receptor. Thus, integrin β1, MLCK, and myosin IIA are factors in the development of resistance to MEK inhibitors. These proteins could provide an opportunity to develop markers and therapeutic targets in a subgroup of triple negative breast cancer (TNBC) that exhibit resistance against MEK inhibition.

## INTRODUCTION

Triple-negative breast cancer (TNBC) refers to tumors that do not express the genes for estrogen receptor (ER), progesterone receptor (PR) and Her2/neu. TNBC patients have been shown to have a poor prognosis compared to non-TNBC patients due to intratumoral heterogeneity [1]. Studies using systematic analysis of functional proteomics revealed that mutant-Ras or Raf drives oncogenic properties of these types of cancers [2]. Indeed, activation of Ras-ERK signaling is associated with increased metastatic risk in breast cancer [3]. Thus, the Ras-ERK pathway is a potential target to reduce tumor progression of TNBC. For this reason, pharmacological agents that target the Ras-ERK pathway have been developed and are under clinical study, including drugs such as Refametinib (BAY 86-9766), and Trametinib (GSK1120212) [4]. Unfortunately, inhibition of the Ras-ERK pathway can result in activation of other oncogenic signaling pathways such as PI3K-AKT, thereby diminishing the therapeutic effects of targeting this pathway due to adaptive resistance of tumor cells [5, 6].

Development of resistance against current therapeutic drugs makes it a challenge to effectively treat cancer patients and thus there is a need for new therapeutic strategies. In several cases, combinatorial inhibition of both the Ras-ERK and PI3K-AKT pathway provides good therapeutic responses compared to individual treatment in clinical trials [7–9]. Although previous research revealed that activation of PI3K-AKT pathway plays a critical role in providing an alternative survival pathway in cancer cells [5–8, 10], the mechanism responsible for the development of resistance to therapeutic drugs in primary and metastatic tumor cells is not fully understood.

In some cases, activation of the PI3K-AKT pathway following inhibition of ERK signaling is associated with different types of cellular receptors such as IGF-1R, EGFR, HER2/3 and PDGFR. In TNBC models, inhibition of MEK results in activation of PI3K-AKT pathway by increased PDGFRβ activity [2]. In contrast to basal-type breast cancer, HER2 and HER3 were defined as critical signaling pathway that mediates activation of AKT following MEK suppression in BT-474 and SKBR-3 respectively [11–13]. In addition, IGF-1R-PI3K was identified as an adaptive resistance signaling pathway against BRAF and MEK inhibitors in melanoma and colon cancer, respectively [7, 14].

We recently reported that the Ras-ERK pathway drives the expression of lung metastasis signature genes as well as alterations in the actin cytoskeleton in MDA-MB-231 LM2 cells that have a preference for lung metastasis [15]. Although inhibition of the Ras-ERK pathway resulted in down-regulation of lung metastasis signature genes, it led to activation of the PI3K-AKT pathway, which drove motility of LM2 cells [15]. Here we extend these studies to include MDA-MB-231 breast cancer cell lines that have a preference for metastasis to brain (BrM2) or bone (BoM2) and investigate the crosstalk between the Ras-ERK and PI3K-AKT pathways. We uncover a critical role for components of the actin cytoskeleton including integrin β1, myosin light chain kinase (MLCK), and myosin IIA to mediate activation of PI3K-AKT and sustained motility when the ERK signaling pathway is inhibited.

## RESULTS

### The Ras-ERK pathway is elevated in MDA-MB-231 cells that metastasize to Bone or Brain and drives the expression of genes associated with metastasis

**Figure 1:**
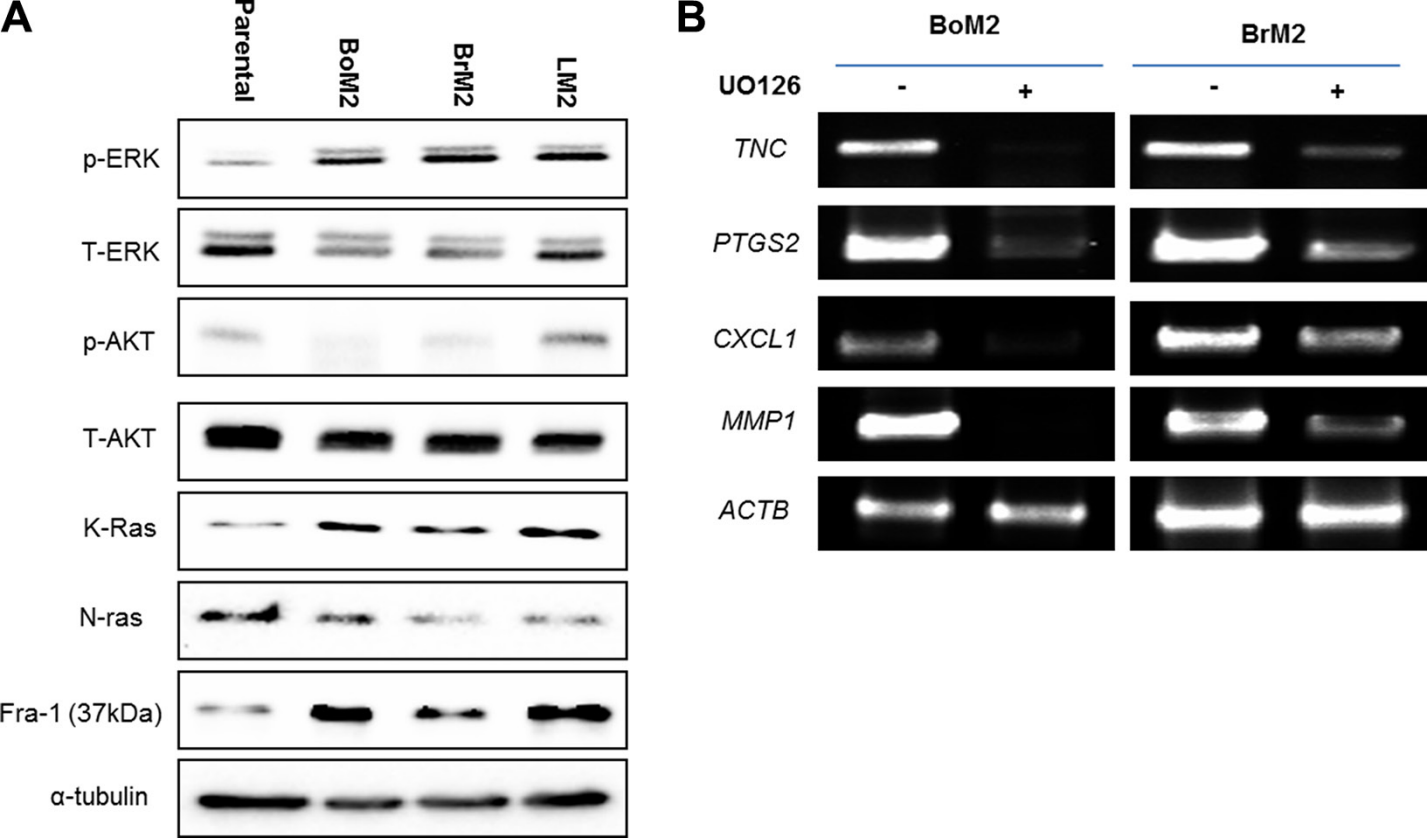
Metastatic TNBC cells have an enhanced Ras-ERK pathway that drives the expression of genes associated with metastasis (**A**) Cell extracts from Parental, BoM, BrM2, and LM2 were immunoblotted to measure levels of indicated proteins among the cells. (**B**) The expression level of indicated lung metastasis signature genes was measured by RT-PCR analysis in BoM2 and BrM2 respectively. Cells were treated with either DMSO or 25 μM UO126 and incubated for 48 hr.

We first performed western blot analyses comparing parental MDA-MD-231 cells and its derivatives that have a preference for metastasis to bone (BoM2), brain (BrM2) or lung (LM2), respectively. BoM2, BrM2, and LM2 were established in the same laboratory for their preference to metastasize to specific tissues [16, 17]. All three metastatic breast cancer cell lines have enhanced Ras-ERK signaling, including a higher amount of K-ras and increased phosphorylated ERK (Figure 1A).

Some of the lung metastasis signature genes are shared with brain and bone metastasis signature genes such as *TNC, PTGS2, CXCL1, and MMP1* [17], Thus, we tested if inhibition of ERK signaling would decrease the expression of several lung metastasis signature genes in BoM2 and BrM2 cells (Figure 1B). Cells were treated with the MEK inhibitor UO126 and analyzed using RT-PCR. Treatment of cells with UO126 resulted in down-regulation of *TNC, PTGS2, CXCL1, and MMP1*. Thus, the Ras-ERK pathway is required for the expression of genes associated with metastasis to brain and bone, in addition to lung.

### Inhibition of Ras-ERK signaling exhibits different effects on cell motility and AKT activation in parental and metastatic cells

**Figure 2:**
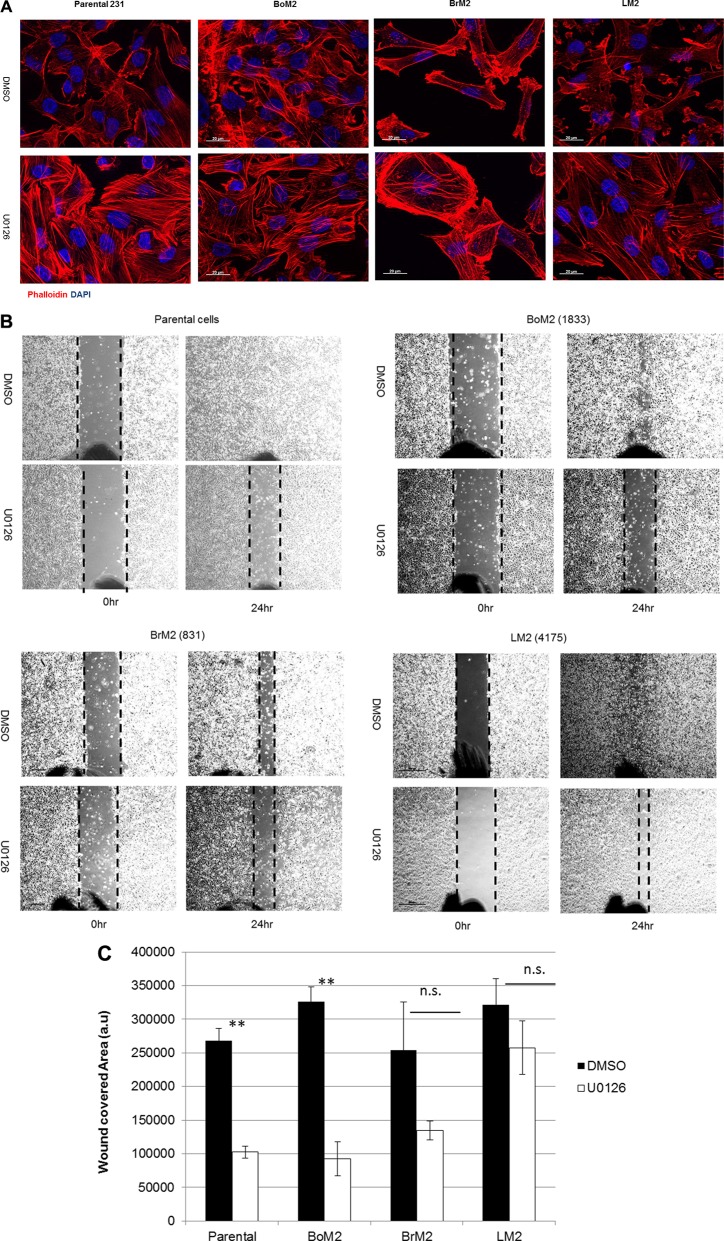
MEK inhibition induces stress fiber formation but differentially modulates motility among parental and metastatic cells (**A**) Parental, BrM2, LM2, and BoM2 cells were treated with either DMSO or 25 μM UO126 and incubated for 48 hr. Cells were fixed with 4% formaldehyde then stained with phalloidin (Red) to visualize actin filaments. Nuclei were stained with DAPI (blue). (**B**) Prior to wound healing migration assay, cells were pre-incubated in 48 hr with either DMSO or 25 μM UO126. Cells in monolayer were scraped with 200 μl tip then discarded the media and fresh media was added together with either DMSO or UO126. (**C**) Measure wounded area by quantification of relative area with three individual experiments. (** < 0.01).

Inhibition of MEK using UO126 increased formation of stress fibers in all metastatic breast cancer cells (Figure 2A). Because the Ras-ERK pathway was responsible for loss of stress fibers and expression of genes associated with metastasis we asked if inhibition of the Ras-ERK pathway would suppress wound healing motility. We performed wound healing migration assay to determine if MEK inhibition would modulate motile behavior of parental, BoM2 and BrM2. MEK inhibition reduced the motility of parental and BoM2 (Figure 2B). In contrast, treatment of BrM2 and LM2 cells with the MEK inhibitor UO126 did not significantly reduce their motility in a wound-healing assay (Figure 2C). These experiments demonstrate that inhibition of the Ras-ERK pathway has distinct effects on the motility of the different types of metastatic derivatives and parental cells. Notably LM2 exhibited insignificant effects in wound-healing in the presence of MEK inhibition, while there was a modest effect on BrM2 cells after treatment with UO126 (Figure 2C).

**Figure 3:**
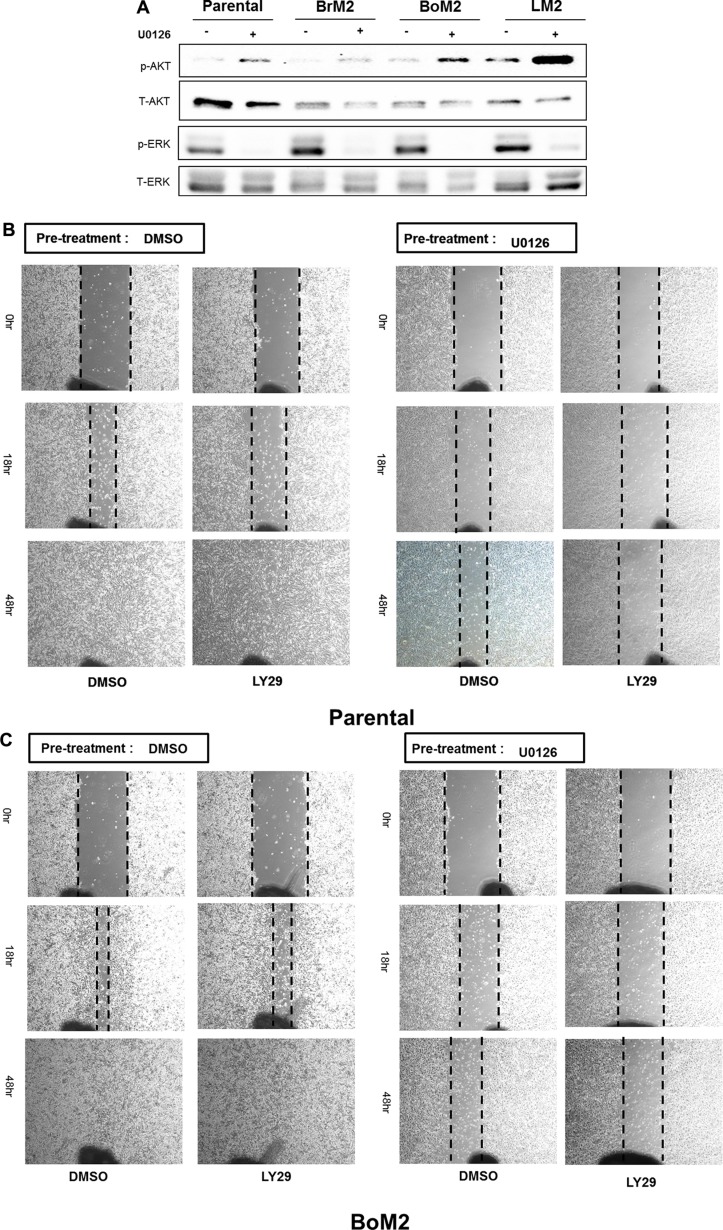

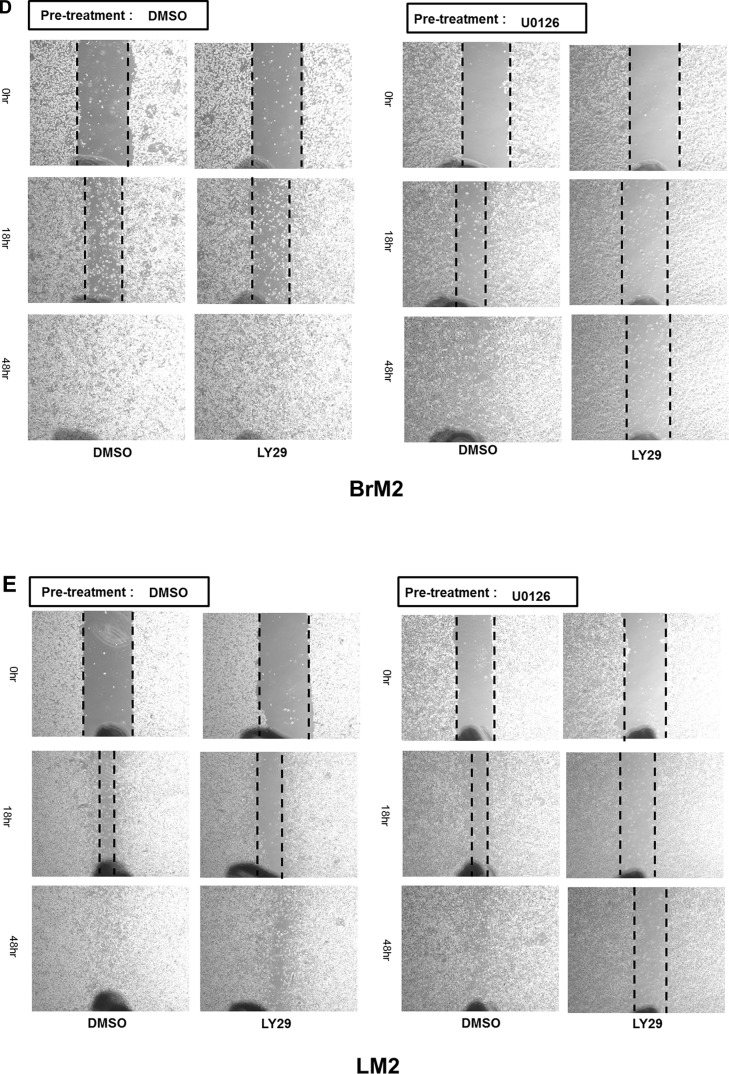
Effects of single and combined MEK and PI3K inhibition on activation of AKT and motility among parental and metastatic derivatives (**A**) Prior to analysis of phosphorylated AKT and phosphorylated ERK by western blot, either DMSO or 25 μM UO126 was added and cells were incubated for 48 hr. Cell extracts were collected with 2× lamelli sample buffer and immunoblotted with indicated antibodies against phosphorylated AKT (S473), AKT, phosphorylated ERK and ERK respectively. (**B**–**E**) Parental and metastatic derivative cells were treated with pharmacological inhibitors of LY294002 and pre-incubated in either DMSO or 25 μM UO126 for 48 hr.

We next determined if MEK inhibition would result in activation of PI3K-AKT signaling after treatment with UO126 among parental and metastatic derivatives (Figure 3A). Inhibition of MEK resulted in higher levels of phosphorylated AKT in BrM2 and LM2 cells, a modest increase in parental cells and undetectable changes in the levels of phosphorylated AKT in BoM2 cells (Figure 3A and 3B). This led us to hypothesize that activation of the PI3K-AKT pathway contributes to motility in LM2 cells following inhibition of the Ras-ERK pathway. Accordingly, we tested the effects of different combination treatments with UO126 and LY294002. Treatment of LM2 cells with the PI3K inhibitor LY294002 did not significantly reduce cell motility. Combinational treatment of LY294002 and UO126 significantly reduced wound healing motility in parental and metastatic breast cancer cells (Figure 3B–3E). However, single treatment with either UO126 or LY294002 did not significantly inhibit wound- healing migration in LM2 and BrM2 cells (Figure 3E).

### Integrin β1 is required for motility and activation of the PI3K-AKT pathway following inhibition of Ras-ERK signaling

**Figure 4:**
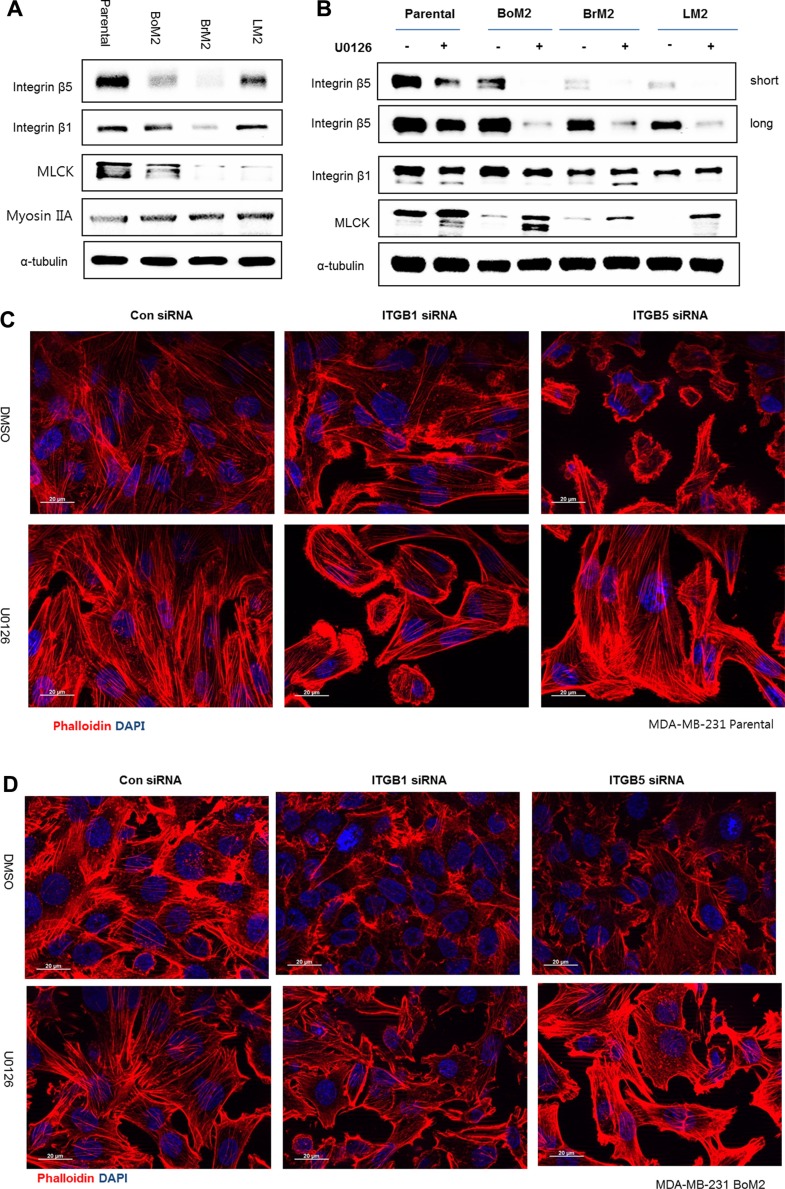

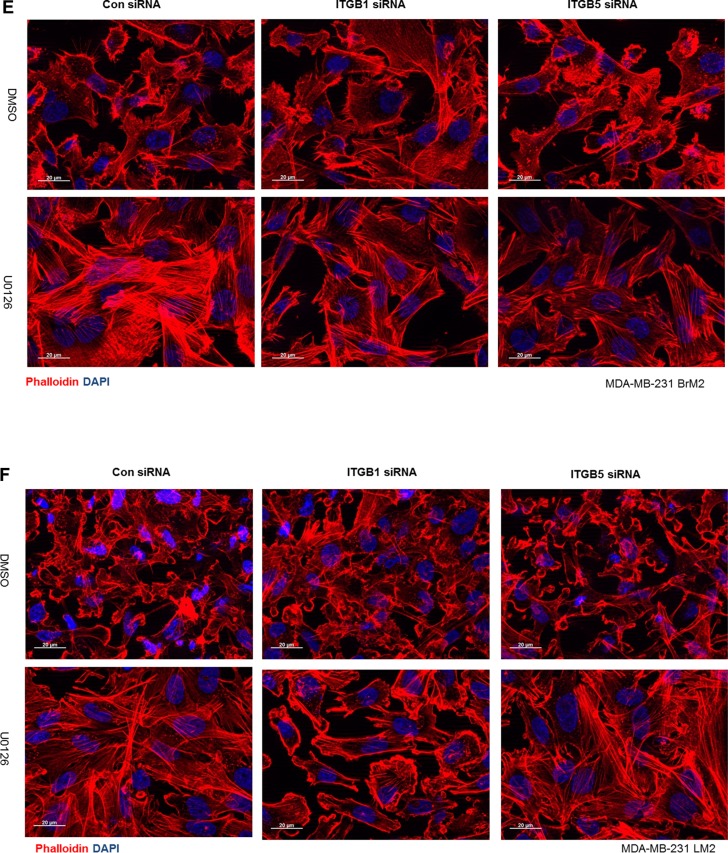
Integrin β1 mediates stress fiber formation and AKT activation following MEK inhibition (**A**) Parental and metastatic cells were extracted with lamelli 2X sample buffer and immunoblotted with indicated antibodies against Integrin β1, Integrin β5 and MLCK. α-tubulin was used for protein loading control. (**B**) Prior to analysis of western blot, cells were incubated with either DMSO or 25 μM UO126. The cell extracts were collected with 2× lamelli sample buffer and immunoblotted with indicated antibodies against Integrin β1, Integrin β5 and MLCK respectively. (**C**–**F**) LM2, Parental, BoM2, and BrM2 cell lines were transfected for 48 hr with control siRNA, Integrin β1 siRNA, or Integrin β5 siRNA and then incubated with either DMSO or 25 μM UO126 for 48 hr. Cells were fixed and stained with phalloidin (Red) to visualize actin filaments. Nuclei were stained with DAPI (blue).

It was reported that integrin β5 is necessary for ventral stress fiber and focal adhesion formation in MDA-MB-231 [18]. We observed inhibition of Ras-ERK signaling led to increased stress fiber formation, which was associated with activation of PI3K-AKT (Figures 2A and 3A). Thus, we asked if integrin β5 was a target of the Ras-ERK pathway and played a role in activation of PI3K-AKT signaling. Compared to parental cells, the metastatic derivatives express lower levels of integrin β5 although levels of integrin β1 are variable (Figure 4A).

Next we asked if inhibition of MEK may increase the levels of integrin β1 or β5 because these proteins are known to play an important role in the organization of the actin cytoskeleton. Unexpectedly, treatment with UO126 resulted in reduced expression of integrin β5 and little effect on the levels of integrin β1 in parental cells and its derivatives (Figure 4B). In addition, silencing integrin β1, and not β5, prevented the restoration of stress fiber organization following MEK suppression (Figure 4C–4E).

**Figure 5:**
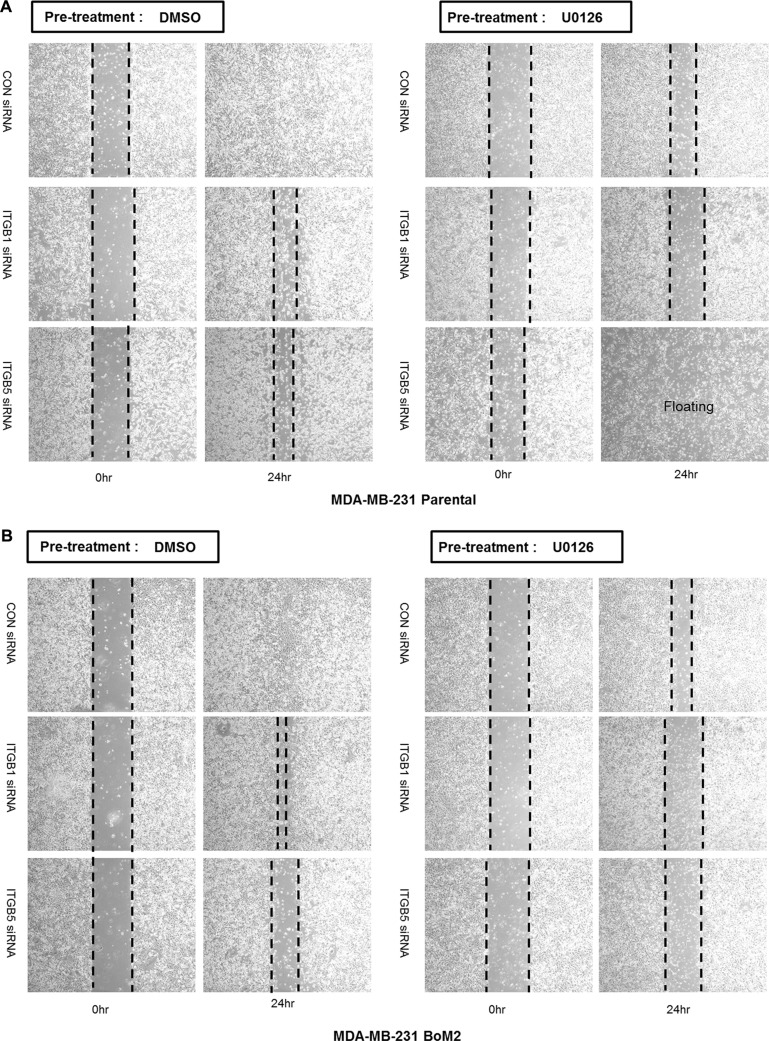

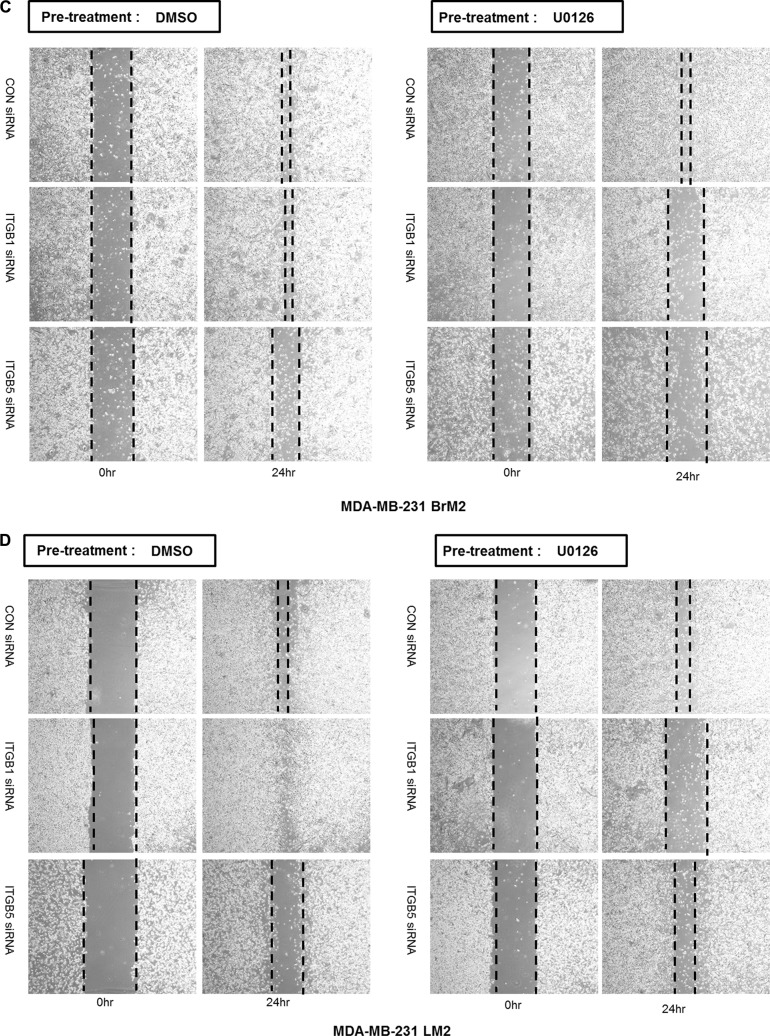

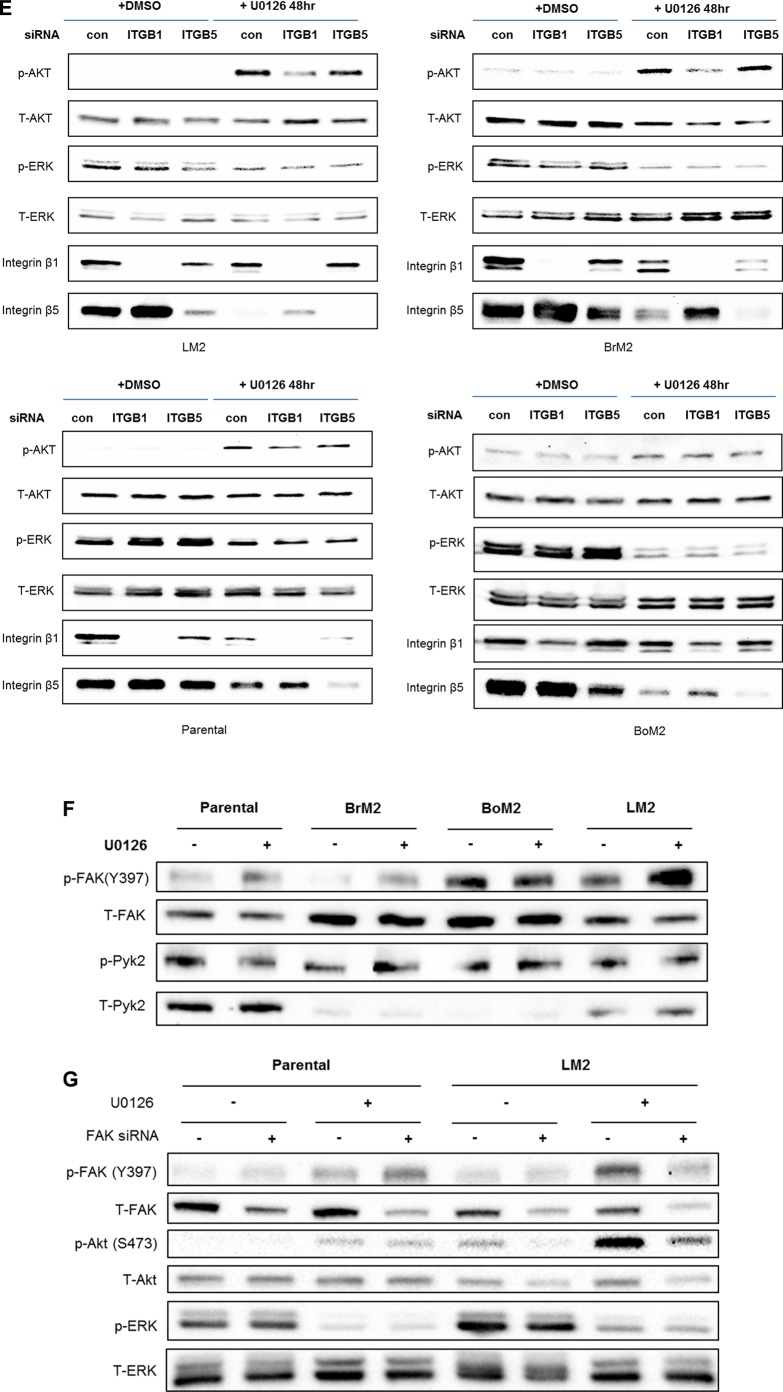

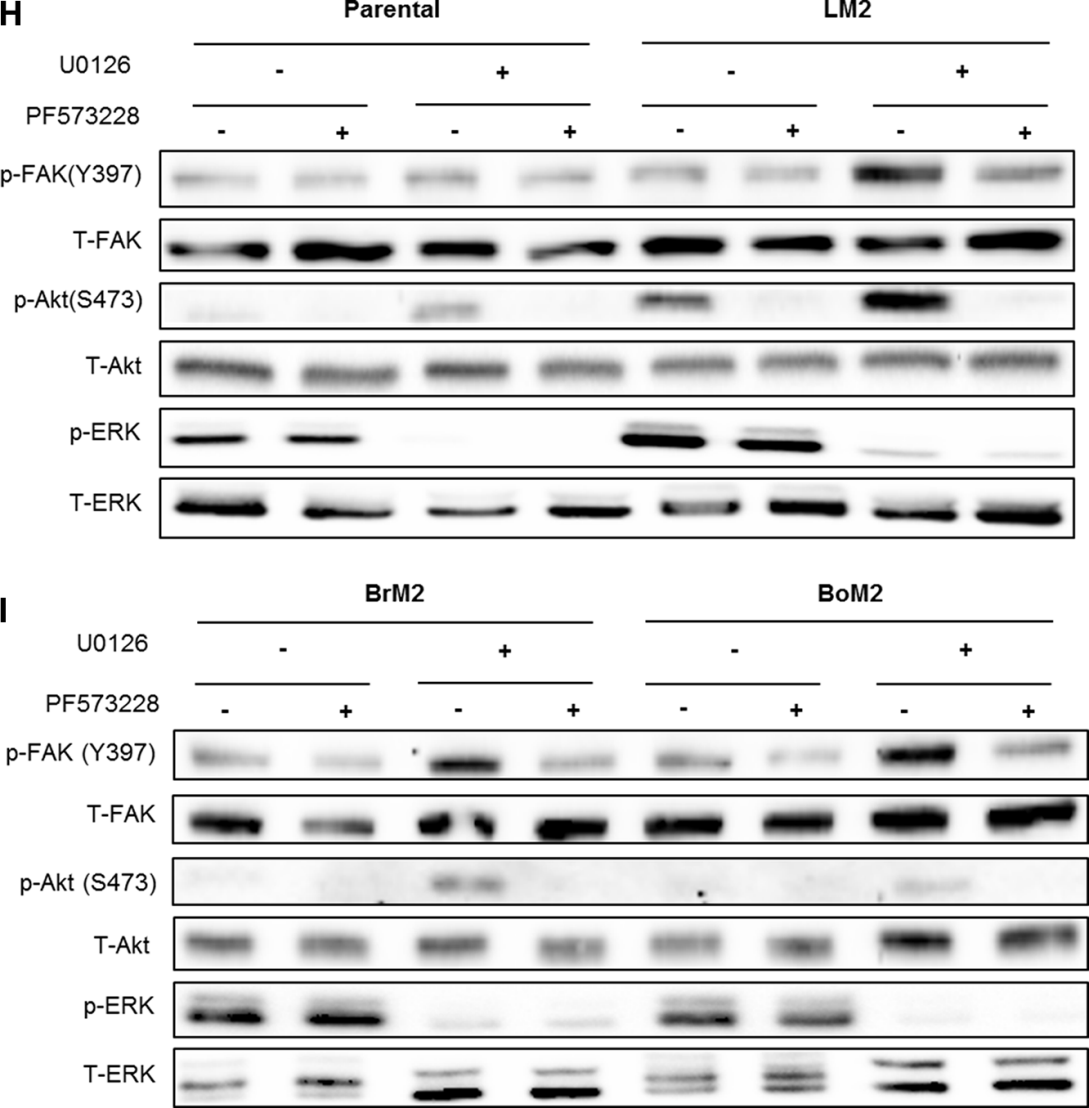
Integrin β1 is required for motility and FAK is required for activation of PI3K-AKT signaling after MEK suppression (**A**–**D**) Integrin β1 or β5 were depleted by siRNA transfection in parental, BoM2, BrM2, and LM2 cells prior to incubation with drug. After transfection of siRNA, cells were treated with either DMSO or 25 μM UO126 for additional 48 hr. Control siRNA was used for negative control. Migration was then measured by wound healing assay. (**E**) Parental, BoM2, BrM2, and LM2 cell lines were transfected for 48 hr with control siRNA, Integrin β1 siRNA, or Integrin β5 siRNA. Cells were then pre-incubated with either DMSO or 25 μM UO126 for additional 48 hr. Cell extracts were immunoblotted to measure the level of phosphorylated AKT. (**F**) Prior to analysis of p-FAK and p-Pyk2 by western blot, either DMSO or 25 μM UO126 was added and cells were incubated for 48 hr. Cell extracts were collected with 2X lamelli sample buffer and immunoblotted with indicated antibodies (**G**) Parental and LM2 cells were transfected for 48 hr with control siRNA, FAK siRNA, or Pyk2 siRNA as 50 nM concentration. Cells were then pre-incubated with either DMSO or 25 μM UO126 for additional 48 hr. (**H**–**I**) Prior to western blot analysis, Parental, BrM2, BoM2 and LM2 cells were treated in combination with both UO126 (25 μM) and PF573228 (10 μM) for 24 hr.

Because integrin β1 is important for the restoration of stress fibers during MEK suppression, we determined if it plays a role in cell motility. Accordingly we tested if down-regulation of integrin β1 or β5 using siRNA would decrease cell motility. In the absence of MEK inhibition, integrin β5 but not integrin β1 was required for motility among metastatic breast cancer cells. By contrast, down-regulation of integrin β1together with MEK inhibition dramatically suppresses motility of metastatic breast cancer cells (Figure 5A–5D).

Because the PI3K-AKT pathway is critical for sustained motility when the Ras-ERK pathway is inhibited, we then investigated if either integrin β1 or β5 are required for activation of AKT following MEK suppression among parental and metastatic breast cancers. Depletion of integrin β1 but not integrin β5 prevented activation of AKT (Figure 5E). These findings suggest that integrin β1 is an upstream target for activating PI3K-AKT pathway and contributes to cell motility following MEK suppression.

FAK and Pyk2 are molecular components that have been reported to be required to activate the PI3K-AKT pathway mediated by integrin signaling [19, 20]. We observed phosphorylation of FAK is increased by treatment of UO126 in parental, BrM2, and LM2 but not BoM2 (Figure 5F). Therefore, we wanted to test if knockdown of FAK by siRNA or pharmacological inhibition may interfere with activation of AKT following MEK suppression especially LM2 (Figure 5G). Both siRNA against FAK or inhibition of FAK using PF573228 dramatically block activation of both FAK and AKT following MEK suppression (Figure 5H–5I). These results indicate that FAK plays a critical role in the activation of the PI3K-AKT pathway mediated by integrin β1 following MEK suppression in metastatic breast cancer cells, especially LM2.

### An intact actin cytoskeleton is required for activation of the PI3K-AKT pathway following inhibition of Ras-ERK signaling

**Figure 6:**
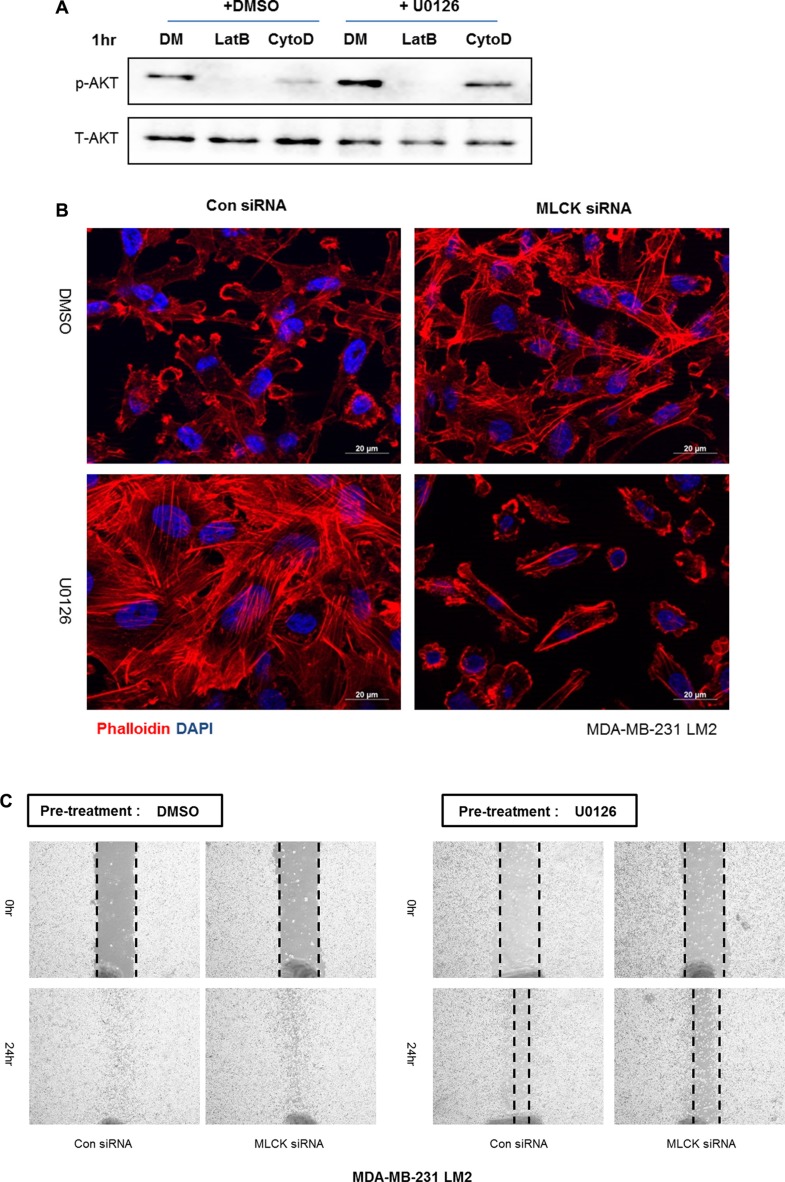

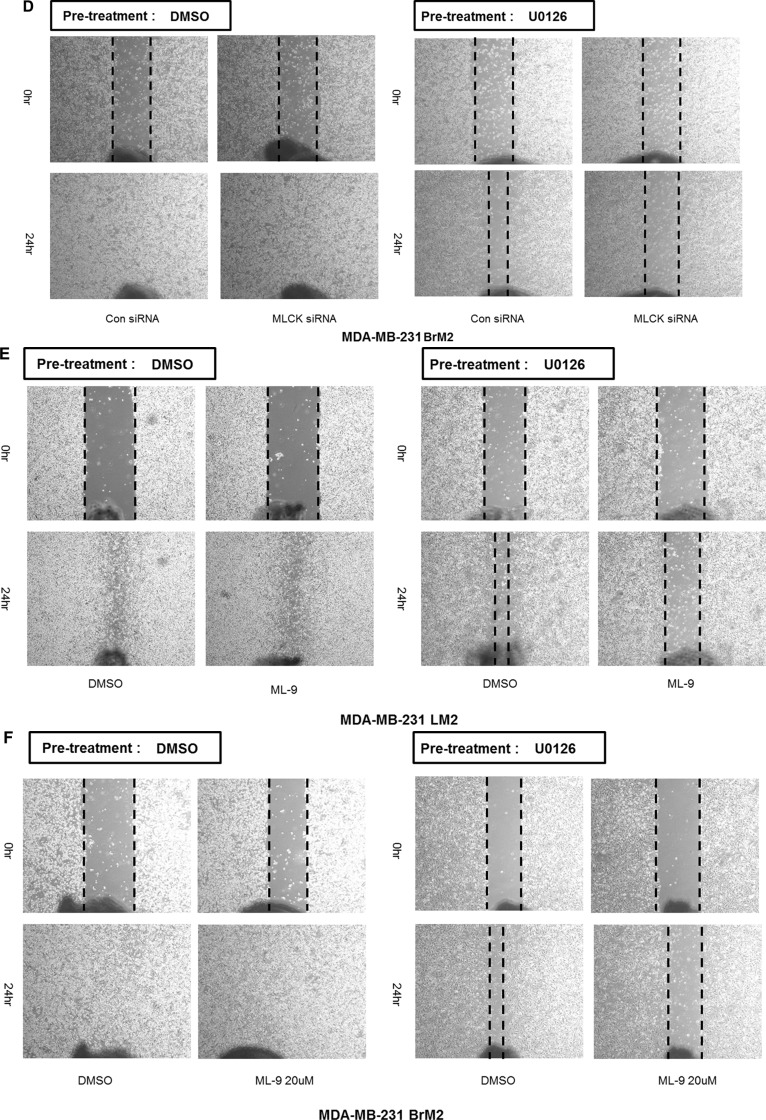

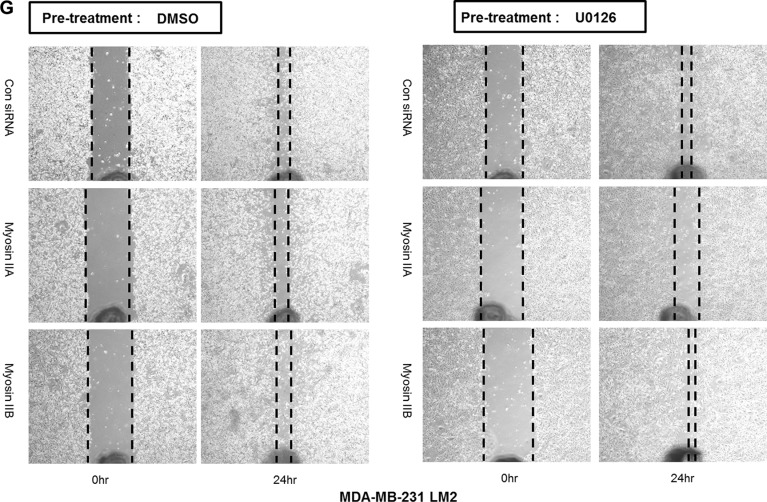
MLCK mediates actin cytoskeleton organization and motility following MEK suppression (**A**) Prior to analysis by western blot, cells were treated with either DMSO or 25 μM UO126 for 48 hr. Cell extracts were collected with 2× lamelli sample buffer and immunoblotted with indicated antibodies against p-AKT(S473) and AKT. α-tubulin was used for protein loading control. Cells were treated with Latrunculin B (5 μM) or Cytochalasin D (1 μM) for 1 hr after pre-incubation of either DMSO or UO126 25 μM for 48 hr. (**B**) Control siRNA or MLCK siRNA was transfected in LM2 cells prior to treating with either DMSO or UO126 (25 μM). After an additional 48hr incubation of drug treatment, LM2 cells were fixed and stained. DAPI and Rhodamine conjugated phalloidin were used for staining nuclei and actin filaments respectively. (**C**–**D**) LM2 and BrM2 cells were transfected with either control siRNA or MLCK siRNA for 48 hr. Cells were then treated with either DMSO or 25 μM UO126 for additional 48 hr. (**E**–**F**) LM2 and BrM2 were pre-incubated with either DMSO or 25 μM UO126 for 48 hr. After pre-incubation, DMSO or ML-9 was then acutely added in fresh culture media. (**G**) LM2 cells were transfected with control, myosin IIA or IIB siRNA and incubated for 48 hr. After transfected siRNA, cells were then pre-incubated with either DMSO or 25 μM UO126 for an additional 48 hr. Cells were scraped by 200 ul tip and added in fresh media with either DMSO or 25 μM UO126 for wound healing migration assay.

Because integrin β1 was associated with sustained motility and activation of PI3K-AKT signaling as well as increased formation of stress fibers following MEK inhibition we asked if the actin cytoskeleton organization was important for activation of the PI3K-AKT pathway. We tested the involvement of actin filaments by directly suppressing actin polymerization or filament formation by treating cells with cytochalasin D or latrunculin B, respectively. Both drugs reduce the activation of AKT with pre-incubation of UO126 in LM2 cells (Figure 6A). Thus the actin cytoskeleton plays a role in activation of PI3K-AKT signaling following inhibition of Ras-ERK signaling.

### MLCK mediates actin cytoskeleton organization and motility following MEK suppression

We next asked what components of the actin cytoskeleton are required for the restoration of stress fibers and sustained motility following MEK suppression. MLCK is known to play a functional role in formation of stress fiber and focal adhesions in various types of normal and cancer cells [21–24]. In agreement with our previous studies in LM2 cells [15], BoM2 and BrM2 contained less levels of MLCK protein compared to parental cells, and inhibition of MEK increased the levels of MLCK (Figure 4B). These data suggested that MLCK could be required for reorganization of the actin cytoskeleton following MEK suppression. Accordingly we tested if MLCK was required for formation of stress fibers and focal adhesions following suppression of MEK by knockdown of MLCK with siRNA. Depletion of MLCK by siRNA prevented formation of stress fibers and cell spreading of LM2 cells following MEK suppression (Figure 6B). In addition, MLCK is also required for stress fiber formation following MEK suppression in parental and other metastatic derivatives including BrM2 and BoM2 (data not shown).

We then asked if MLCK was required for wound healing motility when the Ras-ERK pathway is suppressed. RNAi against MLCK reduces the motility following inhibition of MEK by UO126 (Figures 6C and 6D). Interestingly, knockdown of MLCK alone in the absence of MEK suppression does not inhibit wound healing motility (Figure 6C and 6D). In addition, pharmacological inhibition of MLCK with ML-9 suppress motility of LM2 and BrM2 following MEK suppression (Figure 6E and 6F).

The substrate of MLCK is the regulatory light chain of myosin II. It is also known that integrin clustering requires acto-myosin contractility mediated by non-muscle myosin II motor activity. Two isoforms of myosin II (IIA and IIB) are expressed in MDA-MB-231 cells. It is worth noting that these cells have undetectable levels of myosin-IIC. We then tested if either myosin IIA or IIB is required for motility following MEK inhibition. Interestingly, Myosin-IIA, but not IIB is required for wound healing motility in the presence of UO126 (Figure 6G).

### MLCK and myosin IIA mediates PI3K-AKT pathway following MEK suppression

**Figure 7:**
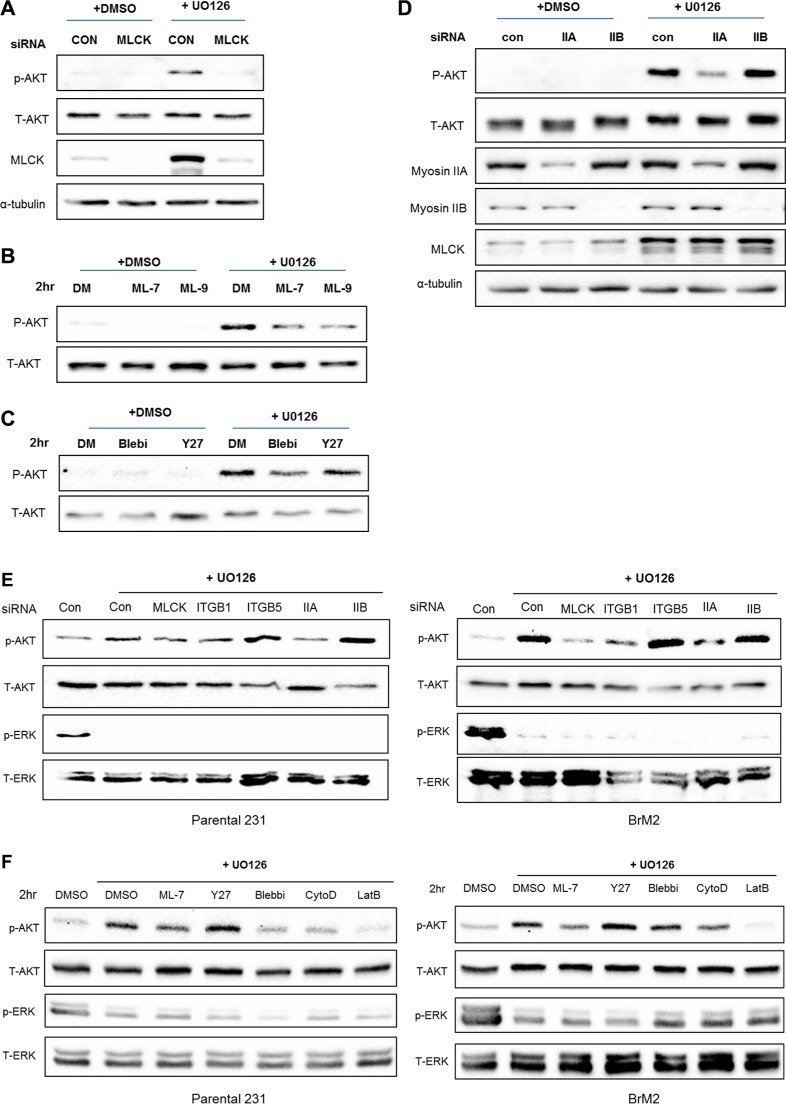
MLCK and Myosin IIA are required for PI3K-AKT activation following MEK suppression (**A**) Control siRNA or MLCK siRNA were transfected in LM2 cells prior to treating with either DMSO or UO126 (25 μM). After an additional 48 hr incubation of drug treatment cells were collected with 2× lamelli sample buffer and immunoblotted with antibodies against phosphorylated AKT, AKT, MLCK. α-tubulin was used for protein loading control. (**B**) LM2 cells were treated with pharmacological inhibitors of MLCK, ML-7 and ML-9 respectively pre-incubated in either DMSO or 25 μM UO126 for 48 hr. (**C**) LM2 cells were acutely treated with Blebbistatin (50 μM) or Y27632 (20 μM) for 2 hr, which is pharmacological inhibitors of myosin II and ROCK respectively pre-incubated in either DMSO or 25 μM UO126 for 48 hr. (**D**) LM2 cells were transfected with either control siRNA or specific myosin II isoform, IIA and IIB respectively. After transfected, cells were incubated with DMSO or 25 μM UO126 for an additional 48 hr. (**E**) Prior to western blot analysis, Parental and BrM2 were pre-treated with UO126 for 48 hr. Then, cells were treated with various actin cytoskeleton disrupting drugs including ML-7 (20 uM), Y27632 (10 uM), Blebbistatin (50 uM), Cytocalasin D (1 uM) and Latruculin B (5 uM) for 2 hr. (**F)** Parental and BrM2 cells were transfected with individual siRNA against MLCK, integrin β1, integrin β5, myosin IIA and myosin IIB. After then Cells were treated with UO126 for 48 hr. AKT activation was measured by immunoblotting with phosphorylated AKT antibody detecting S473 phosphorylation.

As described above, one important regulator of actin filament structure that is up-regulated following inhibition of Ras-ERK signaling is MLCK. Interestingly, MLCK is implicated in activation of integrin β1 [25]. Depletion of MLCK by siRNA knockdown suppresses motility of LM2 and BrM2 cells following MEK suppression (Figure 6), similar to what we observed following integrin β1 depletion (Figure 5). We hypothesized that MLCK plays a critical role of mediating PI3K-AKT activation following MEK suppression. Thus, we tested if inhibiting MLCK function would reduce the activation of PI3K-AKT pathway. Silencing of MLCK by siRNA decreased the activation of AKT following inhibition of MEK (Figure 7A). Furthermore, pharmacological inhibition of the kinase function of MLCK by treatment with either ML-7 or ML-9 reduced phosphorylation of AKT following by MEK inhibition (Figure 7B). These results show that MLCK is required for activation of PI3K-AKT signaling following suppression of Ras-ERK pathway.

In addition to MLCK, non-muscle myosin II is activated by several kinases of that phosphorylate the regulatory light chain of myosin II, including Rho-associated protein kinase (ROCK). Therefore, we tested whether inhibition of acto-myosin contractility by either Blebbistatin or Y27632, the inhibitory drugs against myosin II and ROCK respectively, would reduce activation of PI3K-AKT signaling. Treatment with Blebbistatin reduces phosphorylation of AKT whereas inhibition of ROCK by treatment of Y27632 did not reduce phosphorylation of AKT following MEK inhibition (Figure 7C). In addition, down-regulation of myosin-IIA but not IIB blocks phosphorylation of AKT following inhibition of MEK (Figure 7D). Collectively, these studies suggest that acto-myosin contractility driven by MLCK and myosin IIA is required for activation of PI3K-AKT signaling following suppression of Ras-ERK pathway.

### Activation of PI3K-AKT pathway is independent to EGFR signaling following MEK suppression

**Figure 8:**
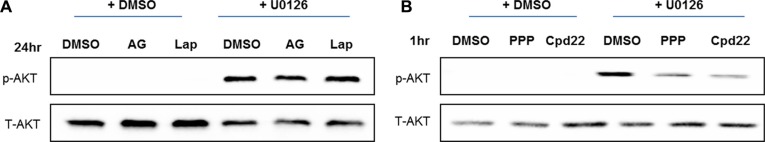
Feedback activation of PI3K-AKT pathway following MEK suppression is independent of EGFR (**A**) LM2 cells were treated with either DMSO or UO126 (25 μM) for 24 hr. Additionally, LM2 were then treated with DMSO, AG1478 (10 μM) or Lapatinib (5 μM) in combination treatment with either DMSO or UO126. After additional 24 hr incubations, cells were collected with 2× lamelli sample buffer and immunoblotted with antibodies against phosphorylated AKT or AKT. (**B**) LM2 cells were treated with either DMSO or UO126 (25 μM) for 48 hr. Additionally, cells were treated with DMSO, PPP (2 μM) and CPD22 (2 μM) respectively combinatorial treatment with either DMSO or UO126. After an additional 1hr incubation, LM2 cells were collected with 2× lamelli sample buffer and immunoblotted with antibodies against phosphorylated AKT or AKT.

It been reported that inhibition of the Ras-ERK pathway mediates feedback activation through various signaling cascade such as HER family receptors [11] and insulin-like growth factor receptor 1 (IGF-1R) [14]. In addition, integrin linked kinase (ILK) is required to activate feedback activation of PI3K-AKT pathway following MEK suppression in glioblastoma cells [26]. Thus, we wanted to identify which pathway might mediate feedback activation of the PI3K-AKT pathway in metastatic breast cancer cells, especially in LM2. We tested if suppression of EGFR signaling may decrease activation of AKT following MEK suppression. Surprisingly, inhibition of EGFR activity by either AG1478 or Lapatinib did not decrease activation AKT following MEK suppression (Figure 8A). We then tested if inhibition of either IGF-1R or ILK suppresses feedback activation of AKT following MEK suppression. Interestingly, both inhibition of IGF-1R and ILK by treatment with PPP and CPD22 dramatically decreases feedback activation of AKT following MEK suppression (Figure 8B). These results are consistent with previous findings that IGF-1R, but not HER families are critical to mediate feedback activation of AKT following K-ras silencing in various K-ras mutant colon cancers [14].

## DISCUSSION

Cancer invasion and metastasis occur during the evolution of a primary tumor, where cells gain the ability to invade and metastasize, resulting in life threatening disease. Elucidating the genes and signaling pathways that mediate metastasis is a critical goal in the development of therapies targeting metastatic disease. To identify genes and signaling pathways that are critical for metastasis here we use the LM2, BrM2 and BoM2 cell models of breast cancer that are derivatives of MDA-MB-231 cells that have a preference for metastasis to lung, brain and bone, respectively. In this paper we demonstrate that an elevated Ras-ERK pathway has a role in regulating actin filament dynamics by disrupting stress fiber formation in all three metastatic derivatives. We also show that the Ras-ERK pathway drives the expression of genes associated with metastasis to lung, bone or brain. Inhibition of the Ras-ERK pathway reduced motility in parental and BoM2 cells but not in LM2 and BrM2 cells. Interestingly, inhibition of the Ras-ERK pathway in BrM2 and LM2 cells resulted in activation of PI3K-AKT signaling that was required for continued cell motility. Furthermore, we identify a mechanism in which integrin β1, MLCK and myosin IIA mediate activation of PI3K-AKT and sustained motility following inhibition of the Ras-ERK in LM2 and BrM2 cells.

### Cross-talk between the Ras-ERK and Ras-PI3K pathway drives motility of LM2 and BrM2 cells

Targeting the Ras-ERK pathway has been studied as a potential therapeutic strategy for cancer patients because it drives various properties of tumor cells. However, targeting the Ras-ERK pathway is known to result in activation of another oncogenic signaling pathway, namely the PI3K-AKT pathway, thereby reducing the effectiveness of therapies targeting ERK signaling. It is important to elucidate the molecular mechanisms involved in the interplay between MEK inhibition and PI3K-AKT activation in primary and metastatic tumor cells.

Here we used MDA-MB-231 breast cancer cell lines that are metastatic to lung (LM2), brain (BrM2) and bone (BoM2) to investigate the crosstalk between the Ras-ERK and PI3K-AKT pathways in organ specific metastatic cancer cells. Although inhibition of the Ras-ERK pathway in parental, LM2, BrM2 and BoM2 cells resulted in formation of stress fibers and focal adhesions, there was no significant reduction on cell motility using wound healing assays in LM2 and BrM2 cells (Figure 2B). Interestingly, inhibition of the Ras-ERK pathway resulted in enhanced activation of the PI3K-AKT pathway in BrM2 and LM2 cells. Recently, it was reported that inhibition of the Ras-ERK pathway results in activation of PI3K-AKT [11, 14, 27]. Although the PI3K pathway is a downstream target of Ras, both parental cells and the metastatic derivatives did not have relatively high levels of phosphorylated AKT (Figure 1A). Thus, although these cells contain a mutant K-ras, there is weak activation of PI3K-AKT signaling. This is in agreement with previous studies that reported MDA-MB-231 cells have almost undetectable levels of phosphorylated AKT [28]. However, inhibition of the ERK pathway resulted in activation of the PI3K-AKT pathway (Figure 3A). Although inhibition of the PI3K pathway alone did not significantly reduce the motility of parental, LM2, BrM2, or BoM2 cells, combination treatment with PI3K and MEK inhibitors significantly reduced wound healing motility (Figure 3B–3D). These data demonstrate that activation of the PI3K-AKT pathway drives motility but only when the Ras-ERK pathway is down-regulated. Taken together, these data indicate that cross-talk between PI3K and ERK signaling pathways are important for the regulation of cell motility.

### Integrin β1 and but not integrin β5 is required to activate AKT to mediate sustained motility following inhibition of Ras-ERK signaling independent of EGFR signaling

Because integrin β5 is necessary for ventral stress fiber and focal adhesion formation in MDA-MB-231, we investigated if it was required for stress fiber formation following inhibition of Ras-ERK signaling [18]. In contrast to our expectation, treatment with UO126 resulted in reduced expression of integrin β5 and insignificant changes in the levels of integrin β1 in parental and metastatic breast cancer cells (Figure 4B). Because integrin β1 was associated with sustained motility and activation of PI3K-AKT signaling as well as increased formation of stress fibers following MEK inhibition we asked if organization of the actin cytoskeleton was important for activation of the PI3K-AKT pathway. Disruption of actin filaments suppressed activation of PI3K-AKT signaling following inhibition of Ras-ERK signaling. These results demonstrate that integrin β1 and components of the actin cytoskeleton are important for PI3K-AKT activation following inhibition of Ras-ERK signaling.

Focal adhesion kinase (FAK) and protein tyrosine kinase 2 (Pyk2) have been reported to be downstream effectors to activate PI3K-AKT pathway mediated by integrins [19, 20]. Our results demonstrate that FAK is required for activation of AKT following MEK suppression in LM2. Furthermore, integrin β1-FAK signaling has not previously been identified to play a role in feedback activation of PI3-AKT signaling mechanisms following MEK suppression in metastatic breast cancer cells.

In some studies, EGFR was reported to be a critical downstream target to activate PI3K-AKT pathway following MEK suppression in MDA-MB-231 [29]. By contrast, EGFR was not involved in feedback activation of AKT following MEK suppression in our study (Figure 8A). Although EGFR inhibitors do not decrease activation of AKT following MEK suppression, both IGF-1R and ILK inhibitors significantly suppress feedback activation of AKT (Figure 8B). It has been shown that ILK can mediate activation of AKT and thereby play key role in tumor progression and metastasis interacting with cytoplasmic domain of integrin β1 [30–32]. Collectively these findings together with our studies suggest that integrin β1-ILK signaling pathway is required for feedback activation of AKT and can function as an alternative pathway, independent of EGFR in metastatic breast cancer cells to stimulate motility following MEK suppression. These results raise the possibility that tumor cells in breast cancer patients will exhibit differential sensitivities to therapies that target EGF receptor family members and therapies against alternative signaling pathways, such as integrin β1-ILK might be an effective target in a subgroup of triple negative breast cancer (TNBC) that exhibit resistance against MEK inhibition.

### Acto-myosin contractility is required to activate integrin β1 and PI3K-AKT signaling

Acto-myosin contractility is suggested as one of mechanisms that increase activity of integrins by clustering [33]. Myosin II-mediated acto-myosin contractility is activated via phosphorylation of the regulatory light chain of myosin II by various kinases and such as ROCK and MLCK. MLCK has been reported to play a critical role in the regulation of integrin β1 activity [24, 25, 34]. This prompted us to hypothesize that MLCK, in addition to integrin β1, was required for PI3K-AKT signaling following MEK suppression to activate acto-myosin contractility via phosphorylation of the regulatory lights chains of myosin II. Consistent with this hypothesis, inhibition of MLCK function by either siRNA or pharmacological drugs decreases activation of AKT and motility following MEK suppression respectively (Figure 7). Furthermore, depletion of MLCK decreased the levels active form of integrin β1 following MEK suppression (data not shown). In addition, inhibition of acto-myosin using blebbistatin also inhibited activation of PI3K-AKT signaling. Collectively these data show that MLCK enhances acto-myosin contractility thereby modulating PI3K-AKT signaling and motility via integrin β1 signaling following MEK suppression. These data demonstrate that MLCK is an essential factor to activate acto-myosin contractility and the motility of metastatic cells via integrin β1 clustering following MEK suppression. Interestingly it was reported that depletion of NMH IIA caused loss of focal adhesion formation, while loss of NMH IIB had no effect [35, 36]. Collectively these data suggest that MLCK and myosin IIA are required for the formation of focal adhesions containing active integrin β1 to mediate AKT activation and sustained motility following inhibition of Ras-ERK signaling.

In summary, the current work provides novel insights into cross-talk between Ras-ERK and PI3K-AKT signaling and the role of the actin cytoskeleton and integrin function in the motile behavior of metastatic cancer cells. The experiments in this study may facilitate the applications of current therapeutic drugs by combinational usage of established drugs or strategies against cancer metastasis. According to our investigations, integrin β1 is required for AKT activation and motility following MEK suppression in a subset of metastatic breast cancer. Indeed, integrin β1 has been reported to play a role in signaling pathways that mediate resistance to clinical therapies [37, 38]. Furthermore, integrin β1 is required for proliferation and motility following MEK inhibition in MDA-MB-231 [39]. In addition to integrin β1, our studies suggest that MLCK and myosin IIA may be novel factors in the development of resistance to MEK inhibitors, and targeting these factors might enhance the clinical efficacy of agents that target the Ras-ERK pathway. These proteins could provide an opportunity to develop novel markers and therapeutic targets of resistance against therapies that inhibit MEK in a subset of TNBC which are refractory current therapies.

## MATERIALS AND METHODS

### Cells and cell culture

Dr. Joan Massagué and Dr. Mi-young Kim generously provided MDA-MB-231 parental, LM2, BrM2 and BoM2. These cell lines were cultured in DMEM high glucose (WELGENE, Korea) supplemented with 10% fetal bovine serum (JR scientific, Woodland, CA, USA), 100 U/ml penicillin and 100 ug/ml streptomycin with L-glutamine (WELGENE, Korea). All cells were cultured at 37°C in a humidified CO2 incubator at 5% as suggested in previous works [16].

### Microscopy and immunofluorescence

Cells were seeded on top of cover glass in 6 well plates. At indicated time periods, cells were fixed with 4% formaldehyde (Sigma-Aldrich, St Louis MO, CA, USA) for 15min. 0.1% Triton-X was used to permeabilize cells. Cells plated onto cover glass were incubated for 30–45 min with indicated primary antibodies and followed incubation of mixture of secondary antibody and phalloidin under parafilm. Fluorescence microscopy observer Z1 (Zeiss) with Apotome2 (Zeiss) was used for observation of cell morphology. Morphology of cells was captured with analyzing software, Axiovision 4.8 (Zeiss).

### Wound healing motility assays

Cells were plated in 6 well plates and grown until 80% confluence. Subsequently, cells were treated with 25 uM UO126 or the same volume of dimethyl sulfide (DMSO) before wounding. After 48 hr incubations, scraping with 200 μl plastic tip across the cell monolayer made the wounds. Location of wounds area marked with dots with marker pen. Immediately scraping monolayer of cells, cell culture media was changed with media containing indicated pharmacological inhibitors. Phase contrast microscope (Eclipse TS100, Nikon) was used for capturing the image during time course (0 hr–40 hr). All experiments were repeated at least three times.

### siRNA knockdown

Cells were seeded into 6 well plates at 30–50% confluence. After 24 hr plating, cells were transfected with siRNA against K-ras (Bioneer, Korea), integrin β1 (Bioneer, Korea), integrin β5 (Bioneer, Korea), Fibronectin (Bioneer, Korea), FAK (Bioneer, Korea), MLCK (Bioneer, Korea), myosin IIA, and myosin IIB (Thermo Scientific) or negative control siRNA (Bioneer, Korea) using Lipofectamine RNAi Max (Invitrogen, Carlsbad, CA, USA) according to manufacturer's protocols. Transfected cells were incubated for additional 96 hr at 37°C in a humidified CO2 incubator at 5% before indicated experiments.

### Western blotting

Whole cell extracts were prepared by lysing cells in lamelli 2× sample buffer containing a cocktail of phosphatase inhibitor, phosSTOP (Rhoche) and protease inhibitors. The total lysate of proteins was separated on 6 to 12% SDS-PAGE by electrophoresis. Proteins were transferred to nitrocellulose membrane, and blocked with 5% powdered skim milk in TBST (50 mM Tris, pH 7.5, 150 mM NaCl, 0.01% Tween). The membrane was then incubated with indicated primary antibodies diluted into 5% BSA in TBST. After primary antibody incubations, membranes were incubated with HRP-conjugated anti-mouse or rabbit IgG secondary antibodies diluted into 5% skim milk in TBST for 1 h. Between each steps, membranes were washed with TBST extensively more than 3 times at 5 min. Proteins were visualized with enhanced chemiluminescence substrate (Thermoscientific). Chemi-doc MP (Bio-rad, Inc., USA) was used to capture images. α-tubulin expression was evaluated as a protein loading control. Protein expression was analyzed by densitometry analysis program, ImageJ Software (National Instituties of Health, USA).

### Antibodies, reagents, chemicals

Antibodies and reagents used in experiments were obtained from: Cell Signaling, pp-MLC2, p-ERK, T-ERK, p-MEK, MEK1, p-Raf, Raf and UO126 (MEK inhibitor); K-ras, H-ras and N-ras, MLC2; Covance, myosin IIA, IIB; Sigma-Aldrich, p-FAK((Y397), FAK, MLCK k-36. p-AKT(Ser 473), AKT, p-ERK(Thr202/Tyr204), and ERK were from Cell Signaling. Antibodies against integrin β1, and integrin β5 were from Santa Cruz. Latrunculin B, Cytochalasin D (Sigma-Aldrich), PF573228 (Tocris), MLCK inhibitor ML-7 (Biomol), MLCK inhibitor ML-9 (Biomol), myosin II inhibitor Blebbistatin (Biomol), ROCK inhibitor Y27632 was used.

### RT-PCR analysis

Total RNA from cells was extracted using a RiboEx (Geneall, Korea) and a Qiagen RNA isolation kit (Qiagen, Valencia, CA, USA) according to manufacturer's instruction. The total RNA concentration was measured by Nanodrop spectrometer (Thermoscientific). Generation of first strand cDNA from total RNA and amplification of this cDNA strand were reacted together using Primer ScriptTM one step RT-PCR kit Ver2 (TAKRA, Japan) and PCR machine (Bio-rad, Richmond, CA, USA) according to manufacturer's instructions. Differently expressed mRNA was separated by 1.2 % agarose gel electrophoresis. Specific primer sequences are as follows:

*MMP-1* fwd, 5′-GATGGGAGGCAAGTTGAAA A-3′; *MMP-1* rev, 5′-CTGGTTGAAAAGCATGAGCA-3′; *CXCL1* fwd, 5′-GAAAGCTTGCCTCAATCCTG-3′; *CXCL1* rev, 5′-CCCTGCCTTCACAATGATCT-3′; *PTGS2* fwd, 5′-TGCTGTGGAGCTGTATCCTG-3′; *PTGS2* rev, 5′-GACTCCTTTCTCCGCAACAG-3′;

*TNC* fwd, 5′-GTCACCGTGTCAACCTGATG-3′; *TNC* rev,5′-TCCCAGAGCCACCTAAGAGA-3′;

*ACTB* fwd, 5′-GCTCGTCGTCGACAACGGCTC-3′; *ACTB* rev, 5′-CAAACATGATCTGGGTCATCTTCTC-3′.

### Statistical analysis

The significance of the experimental results was determined by the Student's *t*-test with Microsoft Excel program.
